# Associations between Orosensory Perception of Oleic Acid, the Common Single Nucleotide Polymorphisms (*rs1761667* and *rs1527483*) in the *CD36* Gene, and 6-*n*-Propylthiouracil (PROP) Tasting

**DOI:** 10.3390/nu7032068

**Published:** 2015-03-20

**Authors:** Melania Melis, Giorgia Sollai, Patrizia Muroni, Roberto Crnjar, Iole Tomassini Barbarossa

**Affiliations:** Department of Biomedical Sciences, University of Cagliari, Monserrato, CA 09042, Italy; E-Mails: melaniamelis@unica.it (M.M.); gsollai@unica.it (G.S.); pmuroni@unica.it (P.M.); crnjar@unica.it (R.C.)

**Keywords:** orosensory perception of oleic acid, *CD36*, PROP genotype and phenotype

## Abstract

Orosensory perception of dietary fat varies in individuals, thus influencing nutritional status. Several studies associated fat detection and preference with *CD36* or 6-*n*-propylthiouracil (PROP) sensitivity. Other studies have not confirmed the latter association. We analyzed the relationship between orosensory perception of oleic acid, two *CD36* variants, and PROP tasting. Thresholds of oleic acid perception were assessed in 64 subjects using a modification of the three-alternative forced-choice procedure. Subjects were classified for PROP taster status and genotyped for *TAS2R38* and *CD36* (SNPs: *rs1761667* and *rs1527483*). Subjects homozygous for GG of the *rs1761667* polymorphism showed higher sensitivity to oleic acid than AA subjects. The capability to detect oleic acid was directly associated with *TAS2R38* or PROP responsiveness. PROP non-tasters had a lower papilla density than tasters, and those with genotype GG of the *rs1761667* polymorphism had lower oleic acid thresholds than PROP non-tasters with genotype AA. In conclusion, results showed a direct association between orosensory perception of oleic acid and PROP tasting or *rs1761667* polymorphism of *CD36*, which play a significant role in PROP non-tasters, given their low number of taste papillae. Characterization of individual capability to detect fatty acids may have important nutritional implications by explaining variations in human fat preferences.

## 1. Introduction

Over the last decade, evidence has been presented of the multiple roles of dietary fatty acids as regulators of energy and lipid metabolism, and their effects on human health and disease outcomes [1]. Therefore, the potential capability to discriminate dietary fatty acids selectively and quantitatively may have important implications for nutritional status and health of individuals. In this context, studies aimed at analyzing fat perception are important to understand how individuals make choices about fat-rich foods in terms of the quality and quantity that they ingest [2].

In humans, orosensory perception of dietary fat clearly depends on multiple factors, including textural and odorant properties, as traditionally thought [3]. Recently, the taste component’s significant involvement in dietary lipid detection has been proposed as a sixth primary sensory quality of the gustatory system [4]. The gustatory contribution to fatty acid perception has been further shown by different studies in which the olfactory component was excluded by stopping nasal airflow, or its texture was disguised by using sonicated emulsions [2,3,5,6]. Long-chain fatty acids seem to be primarily responsible for dietary fat gustatory perception in the oral cavity [7,8], despite dietary lipids being mainly comprised of triglycerides. However, the importance of the hydrolysis of triglycerides via a lingual lipase to release free fatty acids when it comes to the orosensory detection of fat has been shown both in rodents [9] and in humans [5]. The initial stages of the signal transduction mechanisms proposed for the gustatory perception of lipids have been studied in several animal models [10,11], including the interaction of fatty acids with the plasma membrane glycoprotein *CD36* [11,12,13,14,15,16]. In humans, the presence of *CD36* protein in gustatory papillae has been documented [17] as the primary long-chain fatty acid receptor in taste bud cells, and so has its role in the orosensory perception of dietary lipid and fat preference [5,18].

A multitude of variations have been shown for oral sensitivity to fat in humans [6,19,20], and many factors can contribute to them. Among them, three common variants in the *CD36* gene—*rs1761667*, *rs1527483* and *rs3840546*—have been associated with oral sensitivity to and preference for fat (the first two), and obesity (the third) [5,18]. Several pieces of evidence have been reported concerning the effect of the polymorphism *rs1761667* on CD36 protein expression levels, which could explain variations in orosensory perception of fats [21,22,23]. Recently, it has been shown that variations in oleic acid taste sensitivity could be related to variations in general taste sensitivity as indicated by differences in the expression of salivary proteins, such as carbonic anhydrase 6, which have been associated with taste perception [24]. Within this context, it would be of great interest to further explain variability in fat sensitivity by analyzing the associations of the *CD36* variants that affect oral fat perception and preference with other genes known to be involved in taste variability. The genetic ability to taste the bitterness of 6-*n*-propylthiouracil (PROP) is one of best-known examples of taste variability, and has been used as a general index of oral chemosensory perception since it is associated with the perception of a wide range of oral stimuli [25]. The ability to taste PROP is associated with haplotypes of the gene that expresses the PROP-binding bitter receptor, *TAS2R38*, which explains most PROP phenotypic variance [26]. Also, oral sensitivity to PROP is related to expression of specific salivary proteins [27,28]. Moreover, a polymorphism in the carbonic anhydrase 6 gene has been shown to affect PROP sensitivity by acting on cell growth and fungiform papillae maintenance, thus providing an explanation for why PROP super-tasters, who have a high number of papillae, are more responsive to a broad range of stimuli [29].

Given the nutritional value of dietary lipids, the relationship between PROP taster status and perception or liking of fat is of particular interest, and has been extensively investigated, albeit with controversial results [30]. Most studies have shown a direct association between PROP sensitivity and fat perception, and an inverse correlation with the liking of fat: PROP non-taster subjects gave lower taste intensity ratings for linoleic acid [2], had a lower ability to distinguish fat and creaminess in foods [31,32,33,34,35,36], and a higher preference for dietary fat [32,37,38], compared with PROP taster subjects. However, other reports showed no associations between PROP sensitivity and these variables [39,40]. On the other hand, the role of PROP status in salt perception is primarily focused on the analysis of NaCl [41], while evidence suggesting that this phenotype can influence the perception or selection of salty foods is so far lacking. Some studies suggested that PROP related sensory differences may not be limited to taste, but extended to the olfactory system [42], and that PROP status may influence the perception of foods via their aromas or flavors [32,43].

Based on these considerations, investigations on the role of PROP status and *CD36* in fat perception and preferences could better characterize the genetic contribution to fat ingestion and shed a light on potential links to dietary fat nutritional aspects. Here, we analyzed the relationship between the oral threshold for multimodal oral perception to oleic acid, the major liposoluble nutrient in the human diet [44], the two common variants (*rs1761667* and *rs1527483*) in the *CD36* gene, and PROP tasting (genotype and phenotype) as factors that may influence oral perception of dietary fats. Oleic acid orosensory detection thresholds were measured in subjects genotyped for *CD36* and *TAS2R38* polymorphisms and classified for their PROP taster status by having the fat stimulus presented in impregnated filter paper disks.

As mentioned, fats are complex stimuli that provide taste, olfactory, and textural cues, and are further influenced by different physical states (liquid, solid, and semi-solid). These considerations, together with the hydrophobicity of tastants and their high susceptibility to oxidation processes, justify the multiplicity of methods for measuring oral fat perception, all of which have limitations [19,20,32,34,45] such as complicated, lengthy and cumbersome procedures that test subjects must undergo. This fact could represent a factor contributing to the large individual differences that have been reported for oral fat perception, which is why it is important to find an effective, simple and reliable method to be applied in basic research studies [45].

## 2. Experimental Section

### 2.1. Subjects

Sixty-four non-smoking Caucasian subjects (23 males, 41 females, age 27.6 ± 0.85 years) from Sardinia, Italy were recruited according to standard procedures. All were normal weight with a body mass index (BMI) ranging from 18.6 to 25.3 kg/m^2^, had maintained a stable weight in the previous 3 months, and did not follow a diet or take medications that might interfere with taste function. Subjects with extreme scores for restraint and/or disinhibition and/or perceived hunger, assessed by the Three-Factor Eating Questionnaire, were excluded from the study [46]. Normogeusia for four of the basic taste qualities (sweet, sour, salty, and bitter) was verified in all subjects by taste strips (Bunghart Messtechnik, Wedel, Germany). Subjects were informed about the procedure and the aim of the study. All approved and signed an informed consent form. The Ethical Committee of the University Hospital of Cagliari approved the study procedures (Protocol No. 451/09, 15 October 2009; Amendment No. 8, 29 November 2010), which were performed in accordance with the latest revision of the Declaration of Helsinki.

### 2.2. Study Design

All subjects (*n* = 64) were tested in three sessions separated by 1-month periods. They were assessed for PROP taster status in the first two sessions, while in the third session, sensitivity to oleic acid flavor was assessed. All were requested to refrain from eating, drinking (except water), and using oral care products or chewing gum for at least 8 h prior to testing. Women were tested on the sixth/seventh day of their menstrual cycle to avoid oral sensitivity changes due to the estrogen phase [47,48,49,50]. A group of subjects (*n* = 36) were also tested for sensitivity to oleic acid esterified with glycerol (triolein) in a fourth session.

All solutions used for the assessments were prepared the day before each session and stored in a refrigerator until 1 h before testing. Stimuli were presented at room temperature.

### 2.3. PROP Taster Status

Assignment of each subject to a PROP taster group (super-taster, medium taster, or non-taster) was performed using the three-solution test according to Tepper *et al.* [51], which has been validated in several studies [27,28,52,53,54]. Briefly, the taste intensity rating for three suprathreshold PROP (0.032, 0.32, and 3.2 mmol/L) and sodium chloride (NaCl; 0.01, 0.1, 1.0 mol/L) solutions was collected in each subject, by using the Labeled Magnitude Scale [55], which gave subjects the freedom to rate the PROP bitterness relative to the “strongest imaginable” oral stimulus they had ever experienced in their life. Each stimulation was followed by oral rinsing with spring water. The order of presentation of the taste stimuli (PROP or NaCl) in 10 mL samples was reversed in the two sessions. Concentrations were tested in a random order, and the interstimulus interval was set at 60 s. The mean rating of the two replicates was calculated, and functions of perceived taste intensity for PROP and NaCl for each subject were generated from the results [51]. Subjects who gave lower intensity ratings to PROP than to NaCl were classified as PROP non-tasters, those who gave similar ratings to the two stimuli were classified as medium tasters, and those who gave higher ratings to PROP than to NaCl were classified as super-tasters.

Identification and count of the fungiform papillae in a circle area (6 mm in diameter) of the tip of the anterior tongue surface in PROP taster and non-taster subjects were performed according to Melis *et al.* [29].

### 2.4. Oleic Acid Threshold Assessments

**Figure 1:**
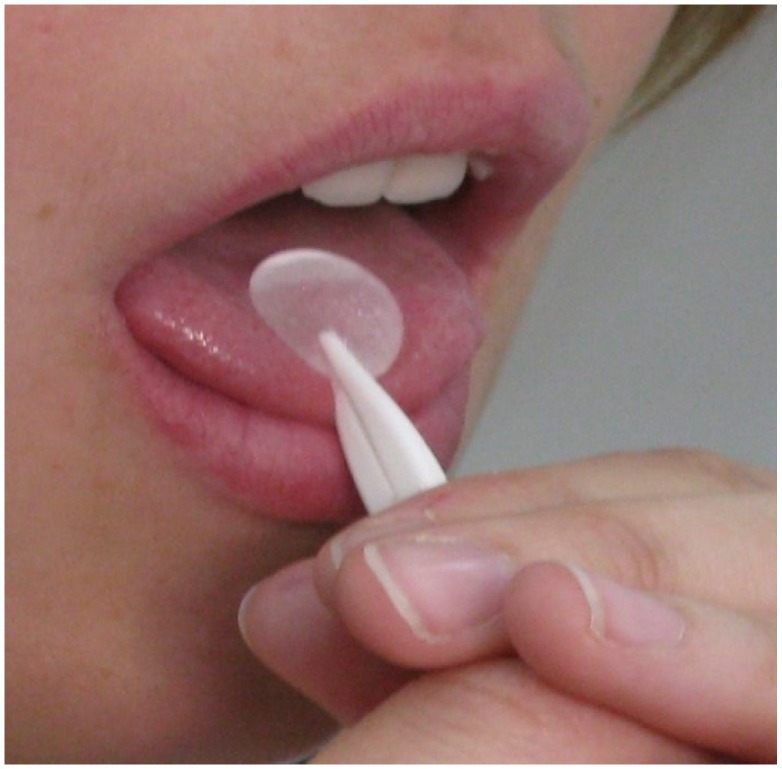
Photograph showing a stimulus presentation in the fatty acid flavor threshold assessment. The stimulation was performed by positioning, on the anterior part of the tongue, the paper disk impregnated with 10 μL of a mixture of oleic acid (or triolein) and mineral oil with the former ranging from 0.0015 to 10 μL (pure).

The threshold for oleic acid multimodal oral perception was assessed in each subject, in the absence of nose clips, by a modification of the staircase method implemented in a three-alternative forced-choice procedure [5], where stimuli were presented to subjects by means of filter paper disks (1.5 cm diameter) (Figure 1). Filter paper disks were impregnated with 10 μL of a mixture of oleic acid and mineral oil, with oleic acid ranging from 0.0015 to 10 μL (pure). Subjects were asked to place the paper disk on the center of their tongue, keep it in the mouth for 10 s and then spit it out. Each subject was presented with three samples: two contained only mineral oil (control) and one the amount of oleic acid under evaluation. They were instructed to savor each disk in order to facilitate the release of the stimulus. The interstimulus interval was set at 60 s. Subjects were instructed to taste the three samples, without swallowing, and to indicate the sample that was different. They rinsed their mouth with deionized water before and after tasting each sample. The procedure continued with the presentation of a new concentration only when the subject reported having no perception. The oleic acid concentration presented was increased after a single incorrect response and reduced after two correct responses in a row. A reversal was considered to have occurred at points where the concentration sequence changed direction. The procedure was terminated when four reversals occurred. The threshold concentration was calculated as the mean value of the four reversals.

### 2.5. Molecular Analyses

Subjects were genotyped for two *CD36* single nucleotide polymorphisms (SNPs), *rs1761667* (G/A) located at the −31,118 promoter region of exon 1A and *rs1527483* (C/T) located at intron 11. DNA was extracted from saliva samples using the QIAamp^®^ DNA Mini Kit (QIAGEN S.r.l., Milano, Italy) according to the manufacturer’s instructions. Purified DNA concentration was estimated by measurements at an optical density of 260 nm. A polymerase chain reaction (PCR) was employed to amplify the *CD36* gene region, including the two polymorphisms. The primers were synthesized by Invitrogen (50 nmol scale, desalted) (Europrim, Invitrogen Cambridge, UK). Molecular analyses were performed using PCR followed by restriction enzyme analysis of the fragments obtained according to Banerjee *et al.* [56], and as briefly described below. To genotype the *CD36* *rs1761667* polymorphism, a 190-bp fragment was amplified with forward 5’-CAAATCACAATCTATTCAAGACCA-3’ and reverse 5’-TTTTGGGAGAAATTCTGAAGAG-3’ primers. DNA was amplified using EuroTaq thermostable DNA polymerase (EuroClone S.p.A., Pero (MI), Italy). Thermal cycles of amplification were carried out in a Personal Eppendorf Master cycler (Eppendorf, Hamburg, Germany). The amplification protocol included an initial denaturation at 95 °C for 5 min, followed by 30 cycles of denaturation at 95 °C for 30 s, annealing at 56 °C for 30 s, and then extension at 72 °C for 30 s. A final extension was carried out at 72 °C for 5 min. The PCR products were digested with the *HhaI* restriction enzyme (Thermo Scientific Inc, Waltham, MA, USA) that recognizes GCG^C site and cut best at 37 °C for 4–16 h. To genotype the *CD36* *rs1527483* polymorphism, the following primer set was used to amplify a 252-bp fragment, the forward 5’-CGCTACAACAATTTTATAGATTTTGAC-3’ and reverse 5’-TGAAATAAAAATAATCTTGTCGATGA-3’ primers. DNA was amplified using EuroTaq thermostable DNA polymerase (EuroClone S.p.A., Italy). The amplification protocol included an initial denaturation at 95 °C for 5 min, followed by 30 cycles of denaturation at 95 °C for 30 s, annealing at 55 °C for 30 s, and then extension at 72 °C for 30 s. A final extension was carried out at 72 °C for 5 min. The thermal cycles of amplification were performed in a Personal Eppendorf Master cycler (Eppendorf, Hamburg, Germany). The amplified samples were digested with the *TaqI* restriction enzyme (Thermo Scientific) that recognizes T^CGA site and cut if incubated at 65 °C for 5–16 h. Digestion products were analyzed by electrophoresis on a 2% agarose gel and the DNA bands were visualized by ethidium bromide staining and ultraviolet light to score the deletion. PCR 50 bp Low Ladder DNA was used as a molecular mass marker (GeneRuler™-Thermo Scientific).

Subjects were also genotyped for three SNPs at base pairs 145 (C/G), 785 (C/T), and 886 (G/A) of *TAS2R38*. This gene expresses the receptor that binds the chemical moiety of the bitter thiourea compounds, such as phenylthiocarbamide or PROP [26]. The three SNPs of *TAS2R38* result in three amino acid substitutions (Pro49Ala, Ala262Val, and Val296Ile) and give rise to two major haplotypes, the dominant taster variant (PAV) and the recessive non-taster variant (AVI), and three rare (AAI, PVI, and AAV). Molecular analyses of *TAS2R38* were performed using PCR techniques followed by the sequencing of the fragments obtained according to Calò *et al.* [52]. Individuals with rare haplotypes were excluded.

### 2.6. Data Analyses

Three-way analysis of variance (ANOVA) was used to compare PROP intensity ratings with NaCl intensity ratings across PROP taster groups (super-tasters, medium tasters, and non-tasters). The Fisher method was used to test genotype distribution and allele frequencies of the two *CD36* SNPs according to PROP status. Threshold differences to oleic acid flavor related to genotypes of the two *CD36* SNPs were evaluated by one-way ANCOVA using papilla density as covariate. One-way ANOVA was also used to compare threshold differences to oleic acid flavor related to PROP tasting (genotype and phenotype), fungiform papilla density (number/cm^2^) between PROP tasters and non-tasters, and PROP bitterness intensity ratings according to *TAS2R38* locus. Two-way ANOVA was used to compare the threshold differences to oleic acid flavor related to genotypes of the two *CD36* SNPs between super-tasters, medium tasters, and non-tasters, or PAV/PAV, PAV/AVI, and AVI/AVI subjects. *Post hoc* comparisons were conducted with the Fisher least significant difference (LSD) test. Statistical analyses were conducted using STATISTICA for WINDOWS (version 7; StatSoft Inc., Tulsa, OK, USA). *p*-Values ≤ 0.05 were considered significant.

## 3. Results

**Figure 2:**
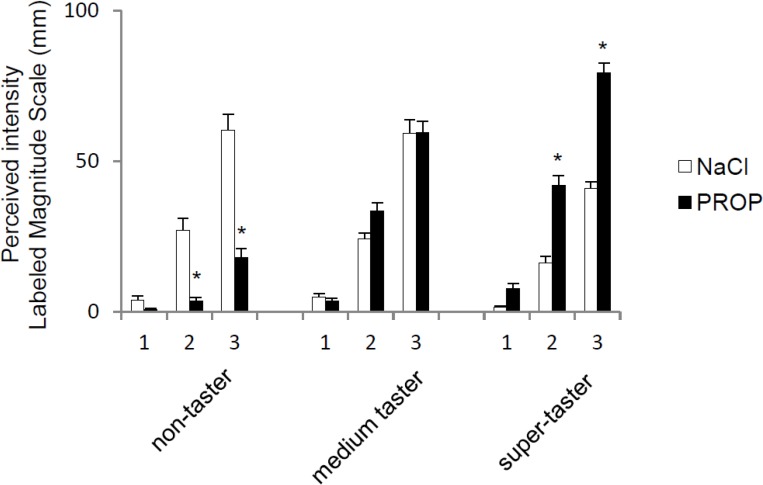
Relationship between perceived taste intensity and stimulus concentration in PROP non-tasters (*n* = 21), medium tasters (*n* = 27), and super-tasters (*n* = 16). All values are mean (±SEM). The numbers 1, 2, and 3 on the x-axis correspond to three NaCl and PROP solutions (NaCl: 0.01, 0.1, 1.0 mol/L) and (PROP: 0.032, 0.32, and 3.2 mmol/L). * Significant difference between PROP and the corresponding sodium chloride concentration (*p* < 0.0001; Tukey *Post hoc* test subsequent three-way ANOVA).

Based on their PROP taster group assignments, 33% of the subjects were non-tasters (*n* = 21); 42% were medium tasters (*n* = 27); and 25% were super-tasters (*n* = 16). ANOVA revealed a significant three-way interaction of taster group × solution type × concentration on the intensity ratings (F_(4,366)_ = 17.587; *p* < 0.0001) (Figure 2). *Post hoc* comparisons showed that non-tasters gave lower intensity ratings to the two highest PROP concentrations compared with the two highest NaCl concentrations, respectively (*p* < 0.0001). Medium tasters gave similar ratings to PROP and NaCl at all concentrations. Super-tasters gave higher ratings to 0.32 and 3.2 mmol/L PROP compared with the two highest NaCl concentrations, respectively (*p* < 0.0001).

Molecular analysis at the three SNPs of the *TAS2R38* locus identified 13 subjects who were PAV homozygous, 31 were heterozygous, and 20 were AVI homozygous. PROP bitterness intensity ratings (3.2 mM) were strongly associated with *TAS2R38* genotypes (F_(2, 61)_ = 57.808; *p* < 0.000001). PROP bitterness ratings were lower in individuals with the AVI/AVI diplotype of *TAS2R38* (16.52 ± 2.72) than in individuals with PAV/PAV (68.01 ± 5.22) and PAV/AVI diplotype (65.94 ± 3.45) (*p* < 0.00011; Tukey *Post hoc* test). No differences in bitterness intensity ratings between PAV/PAV and PAV/AVI subjects were found (*p* > 0.05).

Molecular analysis showed that the observed allele frequencies at each of the two *CD36* SNPs (*rs1761667* and *rs1527483*) were as follows: for the *rs1761667* (A/G) polymorphism, 7 subjects were homozygous AA, 38 were heterozygous, and 19 were homozygous GG, while for the *rs1527483* (C/T) polymorphism, 57 subjects were homozygous CC, 7 were heterozygous, and no subject was homozygous TT. No differences were found among the three PROP taster groups based on the genotype distribution and allele frequency of the two polymorphisms of the *CD36* gene (χ^2^ < 1.358; *p* > 0.061; Fisher method) (Table 1**)**.

**Table 1:** Genotype distributions and allele frequencies of *rs1761667* and *rs1527483* polymorphisms of *CD36* according to PROP taster status.

	Total		PROP Status						*p*-Value *
			Super-Taster		Medium Taster		Non-Taster		
	*n*	%	*n*	%	*n*	%	*n*	%	
*rs1761667*									
*Genotype*									
GG	19	29.69	5	31.25	9	33.33	5	28.80	0.425
AG	38	59.37	11	68.75	15	55.56	12	57.15	
AA	7	10.94	0	0	3	11.11	4	19.05	
*Allele*									
G	76	59.37	21	65.62	33	61.12	22	52.38	0.507
A	52	40.63	11	35.38	21	38.88	20	47.62	
*rs1527483*									
*Genotype*									
CC	57	89.06	16	100	25	92.59	16	76.19	0.061
CT	7	10.94	0	0	2	7.41	5	23.81	
TT	0	0	0	0	0	0	0	0	
*Allele*									
C	121	94.53	32	100	52	96.30	37	88.09	0.072
T	7	5.47	0	0	2	3.70	5	11.91	

* *p*-Value derived from Fisher method (*n* = 64).

**Figure 3:**
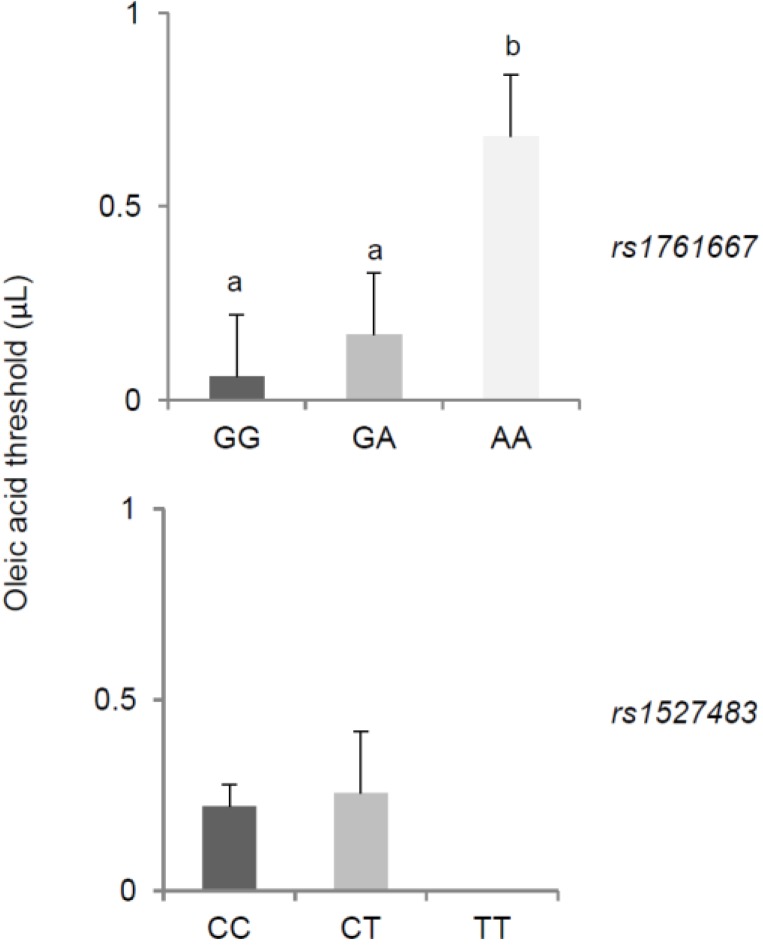
Mean values (±SEM) of the flavor threshold for oleic acid in individuals with genotype GG, GA, and AA of *rs1761667* locus and genotype CC, CT, and TT of *rs1527483* locus of *CD36*. Different letters indicate a significant difference (*p* < 0.033, Fisher LSD test subsequent to one-way ANCOVA).

Mean values (±SEM) of flavor threshold for oleic acid in individuals with genotype GG, GA, and AA of *rs1761667* locus and genotype CC, CT, and TT of *rs1527483* locus of *CD36* are shown in Figure 3. Pairwise comparison subsequent to one-way ANCOVA showed that subjects homozygous for the G-allele of the *rs1761667* polymorphism exhibited a 5-fold lower threshold for oleic acid than homozygous AA subjects (*p* = 0.041, Fisher LSD test) (upper graph). No changes associated with the *rs1527483* polymorphism were found (lower graph).

**Figure 4:**
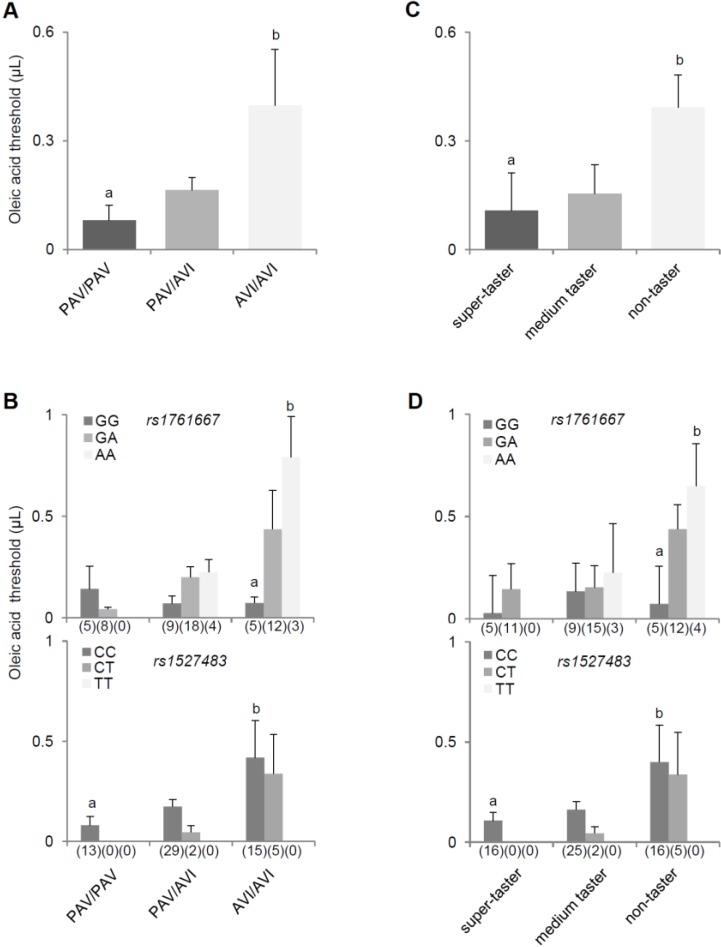
Relationships between flavor threshold to oleic acid, PROP genotype and phenotype, and the two polymorphism (*rs1761667* and *rs1527483*) of *CD36*. (**A**) Mean values (±SEM) of the oleic acid threshold in subjects with genotype PAV/PAV (*n* = 13), PAV/AVI (*n* = 31), and AVI/AVI (*n* = 20) of *TAS2R38*. Different letters indicate a significant difference (*p* = 0.035, Fisher LSD test subsequent to one-way ANOVA); (**B**) Mean values (±SEM) of the oleic acid threshold according to *TAS2R38* and *rs1761667* (upper graph) or *rs1527483* polymorphism (lower graph) of *CD36*. Different letters indicate a significant difference (*p* < 0.037; Fisher LSD test subsequent to two-way ANOVA); (**C**) Mean values (±SEM) of the oleic acid threshold in PROP super-tasters (*n* = 16), medium tasters (*n* = 27), and non-tasters (*n* = 21). Different letters indicate a significant difference (*p* = 0.042, Fisher LSD test subsequent to one-way ANOVA); (**D**) Mean values (±SEM) of the oleic acid threshold according to PROP taster status and *CD36* *rs1761667* (upper graph) or *rs1527483* polymorphism (lower graph). Different letters indicate a significant difference (*p* < 0.048; Fisher LSD test subsequent to two-way ANOVA). The numbers in parenthesis below each bar indicate the number of subjects.

The relationships between the threshold for oleic acid flavor, PROP genotype, and phenotype and the two polymorphisms (*rs1761667* and *rs1527483*) of *CD36* are shown in Figure 4. In particular, Figure 4A shows the mean threshold values (±SEM) for oleic acid in subjects with genotype PAV/PAV, PAV/AVI, and AVI/AVI of *TAS2R38*. *Post hoc* comparison subsequent to one-way ANOVA showed that AVI/AVI subjects exhibited a 5-fold higher oleic acid threshold than PAV/PAV subjects (*p* = 0.035, Fisher LSD). Threshold values for heterozygous subjects were intermediate. Figure 4B shows the threshold values (mean ± SEM) for oleic acid in the same *TAS2R38* genotype groups according to the two polymorphisms of *CD36*. *Post hoc* comparison subsequent to two-way ANOVA highlighted that subjects homozygous AVI/AVI for *TAS2R38* with a genotype homozygous for the G-allele of the *rs1761667* polymorphism had an 11-fold lower threshold for oleic acid than subjects with the same *TAS2R38* genotype (AVI/AVI), but with a homozygous AA genotype of the *rs1761667* polymorphism (*p* = 0.018, Fisher LSD) (Figure 4B, upper graph), and subjects with genotype CC of the *rs1527483* polymorphism of *CD36* who were homozygous for the taster variant in *TAS2R38* (PAV) showed a 5-fold lower threshold than subjects with the same genotype (CC) for this locus of *CD36* who were homozygous for the non-taster variant in *TAS2R38* (AVI) (*p* = 0.037, Fisher LSD) (Figure 4B, lower graph). Figure 4C shows mean values (±SEM) of threshold for oleic acid in PROP super-tasters, medium tasters, and non-tasters. *Post hoc* comparison subsequent to one-way ANOVA showed that PROP non-tasters exhibited a 3.6-fold higher threshold for oleic acid than super-tasters (*p* = 0.042, Fisher LSD). Finally, PROP non-tasters with a genotype homozygous for the G-allele of the *rs1761667* polymorphism of *CD36* had a 9-fold lower threshold for oleic acid than PROP non-tasters with genotype AA of the same locus (*p* = 0.042, Fisher; LSD subsequent to two-way ANOVA) (Figure 4D, upper graph), and PROP super-tasters with genotype CC of *rs1527483* polymorphism of *CD36* showed a 4-fold lower threshold than PROP non-tasters with the same genotype for this locus of *CD36* (*p* = 0.048, Fisher LSD; LSD subsequent to two-way ANOVA) (Figure 4D, lower graph). No other differences relating to the two polymorphisms were found.

The density of the fungiform papillae on the tip of the anterior part of the tongue of PROP non-tasters (52.19 ± 9.45) was lower than that of tasters (92.32 ± 14.27) (*p* = 0.046, one-way ANOVA).

## 4. Discussion

In the light of the evidence collected over the last decade on the nutritional value of dietary lipids [1], and given the wide individual variations in fat consumption and preference [18,57], the present data provide new insights into characterization of individual capability to detect dietary fatty acids in order to identify factors involved in the choice patterns for fat consumption in humans.

Our results show that the paper screening test, a method already used for classifying individuals by PROP taster status [58], is an effective and quick technique and can also be used for assessing oral perception of fatty acids, as well as circumventing the problems related to stimulus hydrophobicity and to the production of homogeneous and stable oil-in-water emulsions. We have been inspired by the elegant approach used by Ebba *et al.* [2], who measured, by means of a scaling method, the chemosensory response to lipid molecules delivered to the oral cavity by edible taste strips. As stated by Prescott, the identification and response to tastants related to food requires the integration, cognitively “constructed”, of functionally united, although anatomically separated, systems (primarily gustatory and olfactory), which can be seen as a functionally distinct sense, the flavor [59]. This is particularly true for the orosensory perception of dietary fats, which clearly depends on activation of taste, olfactory (with aromatic cues) and somatosensory (with textural cues) systems. Therefore, we used threshold measurements to determine individual capability to simultaneously detect the diverse components of orosensory perception of oleic acid, as it happens when it is eaten with food. However, in our study, the contribution of the somatosensory component due to textural cues of the stimulus was eliminated, or greatly minimized, by comparing three paper disks with the same oil content, two only impregnated with mineral oil (control) and one with oleic acid in mineral oil. The threshold values determined with this method were about 10-fold lower (ranging from 0.001 μL to 2.25 μL) than those obtained using texture controlled emulsions [6,19,60].

The major aim of the current study was to determine and characterize factors involved in individual capability to detect dietary fatty acids. A primary finding is that the orosensory perception of fatty acids is directly associated with PROP responsiveness and with the polymorphism *rs1761667* in *CD36*, which seem to play a significant role, mostly in PROP non-taster subjects. In agreement with evidence reported on the effect of this polymorphism on *CD36* protein expression levels, all showing that the homozygous AA condition reduces *CD36* expression [21,22,23], we found that subjects homozygous for the G-allele of the *rs1761667* polymorphism showed a higher capability to detect oleic acid than homozygous AA subjects, who should have a reduced *CD36* expression level, while heterozygous subjects showed intermediate sensitivity. This result confirms recent data obtained from obese subjects [5], even though the threshold values we found were much lower (about 30-fold), probably because we measured the overall response to taste and olfactory components of orosensory perception of oleic acid, in contrast to Pepino *et al.* [5], who completely excluded the olfactory component.

The relationship between PROP taster status and fat perception has been extensively investigated with controversial results [30]. Our data support a direct relationship between fat multimodal oral perception and the genetic ability to perceive the bitter taste of PROP [2]. In fact, we found that the lowest capability to orally detect oleic acid was associated with the lowest responsiveness to PROP (in PROP non-tasters) or with the genotype homozygous for the non-taster variant of *TAS2R38* (AVI/AVI), while the highest sensitivity to oleic acid was associated with the highest responsiveness to PROP (in PROP super-tasters) or the genotype homozygous for the taster variant of *TAS2R38* (PAV/PAV). Several studies showed that PROP non-tasters have a lower density of fungiform papillae than PROP tasters [29,61,62]. Ebba *et al.* [2] hypothesized that the increased ability to taste linoleic acid exhibited by PROP tasters, compared with non-tasters, could be ascribed to the difference in density of fungiform papillae between these two groups. Indeed, our data showed a lower density of fungiform papillae on the anterior part of the tongue of PROP non-tasters, who also exhibit a lower capability to detect oleic acid compared with PROP tasters. In addition, albeit that no differences were found among the three PROP taster groups based on the genotype distribution and allele frequency of the *rs1761667* polymorphism of *CD36*, a comparative analysis indicated that the effect of the *rs1761667* polymorphism of the *CD36* gene on the capability to detect oleic acid was only present in PROP non-tasters or AVI/AVI subjects. In fact, PROP non-tasters (or AVI/AVI subjects) with a genotype homozygous for the G-allele of the *rs1761667* polymorphism had a lower oleic acid threshold than PROP non-tasters with the homozygous AA genotype. Instead, no effects of this polymorphism of *CD36* on oleic acid perception were found in PROP taster subjects. This finding suggests that a high expression level of *CD36* in gustatory cells seems to be a determining factor for detecting dietary fat only in subjects who have a low density of taste papillae.

Recent data have shown that a minor allele of *rs1527483* polymorphism of *CD36* was associated with increased ratings of fat content in salad dressings in African Americans [18]. The very low frequency that we found for the T-allele may explain why no change in capability to detect oleic acid orally, in relation to the *rs1527483* polymorphism, was found in our population sample. On the other hand, the higher threshold exhibited by PROP non-tasters (or AVI/AVI subjects) with a genotype homozygous for the *C*-allele of the *rs1527483* polymorphism of *CD36*, with respect to super-tasters (or PAV/PAV subjects) with the same genotype for this locus of *CD36*, may be due to the difference in papilla density between the two PROP taster groups.

## 5. Conclusions

The present study provides evidence that the paper screening test is a quick, easy, and low-cost method for assessing the orosensory perception of dietary fats. In addition, our findings extend our knowledge about the characterization of individual capability to detect dietary fats in the oral cavity by showing that the polymorphism *rs1761667* in *CD36*, which influences protein expression levels, plays a crucial role mostly in PROP non-tasters, given their low number of taste papillae. A better understanding of individual orosensory capability could lead to the recognition of the wide variation in human fat preferences and consumption patterns, and thus may have important implications for the nutritional status and health of individuals.
